# An Initial Survey on the Prevalence of Group B *Streptococcus* (GBS) among Yemeni Pregnant Women in Sana'a City

**DOI:** 10.1155/2022/6279343

**Published:** 2022-10-18

**Authors:** Ibrahim Al-Subol, Maha Abdul-Aziz, Abdullah A. Almikhlafy, Talal Alqahtani

**Affiliations:** ^1^Department of Medical Laboratory, Microbiology, Faculty of Medicine & Health Sciences, University of Science and Technology, Sana'a, Yemen; ^2^Department of Obstetrics and Gynecology, Faculty of Medicine & Health Sciences, University of Science and Technology, Sana'a, Yemen; ^3^Department of Community and Family Medicine, Dean of Faculty of Medicine & Health Sciences, University of Science and Technology, Sana'a, Yemen; ^4^Department of Health Sciences, Microbiology, Faculty of Medicine & Health Sciences, University of Science and Technology, Sana'a, Yemen

## Abstract

**Background:**

Infection with group B Streptococcus (GBS) is still a neonatal life-threatening illness, especially in developing countries such as Yemen.

**Objective:**

This study was aimed at determining the vaginal colonization rate and antibiotic susceptibility pattern of GBS among Yemeni pregnant women.

**Methods:**

We conducted a cross-sectional study over a four-month period involving 210 pregnant women at the 35th to 39th gestational weeks who visited Gaza medical center in Sana'a city, Yemen. The collected vaginal swab specimen was inoculated in the Todd-Hewitt enrichment broth and incubated for 24 h and then subcultured on a 5% human blood agar plate. All positive cultures identified as GBS were subjected to antibiotic susceptibility tests using the disk diffusion method.

**Results:**

Out of 210 recruited pregnant women, 23 (10.95%) were GBS vaginal carriers. All GBS isolates were sensitive to penicillin, ampicillin, levofloxacin, cefotaxime, and vancomycin.

**Conclusion:**

Based on the study's results, approximately eleven out of every 100 pregnant women in Sana'a city are vaginally colonized by GBS. Beta-lactam antibiotics remain the drug of choice to treat and prevent GBS infections. A prenatal screening policy is urgently needed for Yemeni pregnant women.

## 1. Introduction

On January 1, 2016, the globe formally started implementing the United Nations sustainable development goals (SDGs). One of these 17 goals is enhancing mother, neonate, and child health and planning to end mother and child deaths from preventable diseases [1]. To achieve these goals, neonatal fatalities in each nation should be about 12 per 1000 live births by 2030 [2, 3]. Despite the falling in maternal mortality with substantial variation among countries [4, 5], it is still high especially in developing countries. In 2017, over 295 thousand women died while pregnant or after giving birth, and 94% of these avoidable fatalities occurred in poor and middle-income countries [6]. In low- and middle-income nations, infectious diseases continue to rank among the leading causes of preterm birth and newborn encephalopathy [7, 8], which are major causes of neonatal death and survivors' mental disorders [9–12]. *Streptococcus agalactiae* or group B Streptococcus (GBS) is spherical-shaped (coccus) Gram-positive pathobiont bacteria that is often found in the reproductive and gastrointestinal tracts of healthy women, and it can cause serious maternal and neonatal infections [13, 14]. Worldwide, there is a considerable variation in the prevalence of GBS maternal colonization, ranging from high (35%) in the Caribbean to a much lower prevalence (13% and 11%) in Southern and Eastern Asia, respectively [15]. At the regional level, the prevalence of GBS maternal colonization was 19.5% in Jordan [16], 15% in Saudi Arabia [17], 11.3% in Egypt [18], and 10.1% in the United Arab Emirates [19]. Infants born to mothers who are vaginally colonized by GBS are at high risk of acquiring bacteria and may develop life-threatening neonatal meningitis, pneumonia, and septic shock, and neurological deficits may result among survivors [20–23]. Yemen is one of the least developed nations, and its people are vulnerable to a variety of illnesses, including neonatal infections. Up to date, no data regarding the prevalence of GBS among Yemeni pregnant women has been published. Therefore, this study was aimed at estimating the prevalence and antibiotic susceptibility pattern of GBS among pregnant women in Sana'a city, Yemen.

## 2. Methodology

### 2.1. Study Design, Population, and Setting

We conducted a 4-month cross-sectional study (from June to September 2019) to determine the prevalence of GBS maternal colonization and its antibiotic susceptibility profile among Yemeni pregnant women who attended the Gaza medical center in Sana'a city. Pregnant women who were not within the range of 35^th^-39^th^ gestational weeks, on antibiotic therapy, diabetics, and those who had urinary tract infections were excluded.

### 2.2. Sample Size

The sample was calculated using OpenEpi.com (an open-source web tool) based on an expected prevalence of 15% (according to other regional previous studies) at a confidence level of 95% and an accepted marginal error of 5%. Thus, the calculated sample was 196 and our study involved 210 participants.

### 2.3. Specimen and Data Collection

After the participant consent, a trained nurse carried out vaginal swabbing according to CDC recommendations [24]. The collected specimens were inoculated into Amies transport medium, properly labeled, and transferred immediately to the microbiology laboratory. A structured questionnaire in Arabic that includes information regarding age, education level, previous gestations, previous miscarriages, previous antibiotic use, diabetes, and other chronic diseases was used via face-to-face interview for each participant.

### 2.4. Bacterial Isolation and Identification

The collected specimens were inoculated into the Todd-Hewitt broth containing gentamicin (8 *μ*g/mL) and nalidixic acid (15 *μ*g/mL) and incubated at 37°C for 24 h. In the next day, the incubated Todd-Hewitt broths were subcultured on 5% human blood agar plates and incubated at 37°C for 24 h. The isolated pure colonies were identified as GBS by the following criteria: colony morphology, Gram staining, hemolysis, catalase, and CAMP test [25].

### 2.5. Antibiotic Susceptibility Testing

Antibiotic susceptibility testing for penicillin (10 U), ampicillin (10 *μ*g), levofloxacin (5 *μ*g), cefotaxime (30 *μ*g), clindamycin (2 *μ*g), vancomycin (30 *μ*g), and tetracycline (30 *μ*g) was performed for all GBS isolates using the disk diffusion method on the Mueller-Hinton agar containing 5% human blood according to CLSI guideline [26].

### 2.6. Data Analysis

The results were tabulated and analyzed by IBM SPSS Statistics version 23 for Windows® (IBM Corp., Armonk, NY, USA). Descriptive statistics included frequencies and cross-tabulation; however, the significance of differences was tested by chi-square and Fisher's exact tests. The significance level (*P* value) of less than 0.05 was considered significant.

### 2.7. Research Ethics

The approval to conduct this study was obtained from medical research ethics committee of the University of Science and Technology, NO (ECA/UST191).

## 3. Results

The mean age of 210 participant pregnant women was 26.14 ± 5.28 years ranging from 16 to 38 years. The results of this study showed that 23 pregnant women (10.95%) with 95% CI (6.95 to 14.95%) were GBS vaginal colonized. The mean age of pregnant women with positive GBS vaginal colonization was 26.47 ± 6.5 years, whereas it was 26.1 ± 5.12 years for the rest negative participants. The mean gestational age of pregnant women with positive GBS vaginal colonization was 37.17 ± 1.34 weeks, whereas it was 36.91 ± 1.08 weeks for the rest negative participants, and therefore, there was no correlation between participants' age or gestational age and GBS vaginal colonization. Statistical analysis of the variables (gravida, previous abortions, and educational level) showed that there were no statistical significant differences (*P*˃0.05) between different groups of participants as shown in (Table 1).

All 23 GBS isolates were undergone antibiotic susceptibility testing which showed that all isolates were sensitive to penicillin, ampicillin, levofloxacin, cefotaxime, and vancomycin, while the sensitivity to clindamycin and tetracycline decreased to be 82.8% and 30.5%, respectively (Table 2).

## 4. Discussion

At the community level in Yemen, no research has been carried out to determine the frequency and burden of neonatal infections. According to United States Agency for International Development (USAID) estimates, Yemen still has high under-5 children, infants, and newborns mortality rates (59.6, 45.7, and 28.1 deaths per 1,000 live births, respectively), with an essential portion of this mortality being caused by infectious diseases [27]. Our recent study reported a high proportion of culture-confirmed neonatal sepsis, accounting for two-thirds (77.38%) of neonates admitted to the referral hospitals in Sana'a city [28].

Although prenatal screening for pregnant women is important and beneficial, no official Yemeni guidelines regarding GBS in pregnant women have been established. This cross-sectional study was conducted on 210 Yemeni pregnant women to investigate the prevalence of vaginal colonization by GBS for the first time in Yemen. The present study revealed that 10.95% of Yemeni pregnant women were vaginally colonized by GBS. Given the absence of prenatal screening procedures for Yemeni pregnant women and the resulting lack of access by colonized mothers to antibiotic prophylaxis, this colonization rate entails significant risks. This result is consistent with the findings of several similar studies from developing countries where the GBS vaginal colonization ranged from 10% to 15%. For instance, it was 15% in Bangladesh [29], 10.4% in Ethiopia [30], and 14% in Cameroon [31]. However, GBS vaginal colonization in other countries was reported to be less than that of ours. For example, it was 8.2% in China [32], 7.6% in Saudi Arabia [33], and 2% in India [34], whereas higher colonization rates were reported in other countries, such as 26% in Brazil [35], 19.5% in Jordan [16], and 30.9% in South Africa [36]. The variation of colonization rates among different studies may be attributed to several factors, such as different specimen collection sites (vaginal, rectal, or both), different sample sizes, different personal hygiene practices, different sexual behaviors, antibiotic use, religion and culture beliefs, and different ways to isolate bacteria. The low prevalence in our study may be attributed to the fact that we were unable to persuade the participant to allow us to take a rectal swab. In our study, the relationship between variables (gravida, previous abortions, and educational level) and GBS vaginal colonization was insignificant (*P*˃0.05) (Table 1). This finding conforms to other research findings, such as [13, 16, 37, 38]. Regarding antibiotic resistance, many earlier studies have been performed to find out the sensitivity of GBS to various antibiotics [24, 39, 40]. Our results showed that all isolates were sensitive to penicillin, ampicillin, levofloxacin, cefotaxime, and vancomycin. These findings are close to the findings of other studies, such as [41] done in Nigeria, [42] in Saudi Arabia, and [30] in Ethiopia. Generally, penicillin is yet the drug of choice for prophylaxis and treatment against GBS colonization and infections. Thus, our result is consistent with other research results concerned with GBS sensitivity to penicillin [30, 39, 42, 43], whereas our result is inconsistent with the Ethiopian study result where the highest resistance was observed against penicillin [44]. Women who were penicillin-allergic clindamycin is recommended for GBS prophylaxis during labor [24]. During the last 10 years, GBS strains had exhibited resistance to other antibiotics, including erythromycin and clindamycin [45]. Clindamycin and/or erythromycin resistant GBS has already been notified earlier, ranging from 0.7% to 51.3% for erythromycin and from 1.7 to 50% for clindamycin [46, 47]. In the current study, the GBS clindamycin resistance level was 8.6%, and this is close to clindamycin resistance level (5.1%) in Saudi Arabia [42]. The different antibiotic sensitivity patterns of GBS among different studies may be attributed to different antibiotic practices and different mutated GBS strains.

## 5. Conclusion

In Yemen where prenatal screening is not a routine besides boor awareness of the importance of antibiotic intrapartum prophylaxis, 11% of Yemeni pregnant women are GBS vaginal colonized. Fortunately, in our region, all GBS isolates were sensitive to beta-lactam antibiotics; thus, penicillin remains the first choice for treatment and prophylaxis at the prenatal period of mothers who carry these bacteria and are not penicillin allergic. From the context of what was stated in the results, we recommend an official routine prenatal screening for Yemeni pregnant women in order to take appropriate preventive measures and prevent potentially life-threatening infections of newborns.

## 6. Limitation

One limitation of our study was that the GBS isolates were not subjected to serotyping due to the lack of the required GBS serotyping kit in the Yemeni market.

## Figures and Tables

**Table 1:** Relationship between independent variables and GBS vaginal colonization rate among Yemeni pregnant women in Sana'a city.

Variables		Vaginal colonization				Total	*P* value
		Yes		No			
		*N*	%	*N*	%		
Gravida	0	2	18.20%	9	81.80%	11	0.517
	1	5	12.50%	35	87.50%	40	
	2	3	5.80%	49	94.20%	52	
	3 or more	13	12.10%	94	87.90%	107	
Abortions	No	17	11.30%	134	88.70%	151	0.82
	Yes	6	10.20%	53	89.80%	59	
Educational level	Illiterate	8	14.80%	46	85.20%	54	0.565
	Preparatory	7	8.60%	74	91.40%	81	
	Secondary	8	11.60%	61	88.40%	69	
	University	0	0.00%	6	100.00%	6	

**Table 2:** Antibiotic susceptibility profile of the 23 vaginal GBS isolates from Yemeni pregnant women in Sana'a city.

Antibiotic agent	Antibiotic susceptibility pattern		
	No. (%) of isolates		
	S	I	R
Penicillin G 10 IU	23 (100%)	0 (0%)	0 (0%)
Ampicillin 10 *μ*g	23 (100%)	0 (0%)	0 (0%)
Levofloxacin 5 *μ*g	23 (100%)	0 (0%)	0 (0%)
Cefotaxime 30 *μ*g	23 (100%)	0 (0%)	0 (0%)
Vancomycin 30 *μ*g	23 (100%)	0 (0%)	0 (0%)
Clindamycin 2 *μ*g	19 (82.80%)	2 (8.60%)	2 (8.60%)
Tetracycline 30 *μ*g	7 (30.50%)	5 (21.70%)	11 (47.80%)

S = sensitive, I = intermediate, R = resistant.

## Data Availability

The datasets used and analyzed during the current study are available from the corresponding author on reasonable request.
